# Supplementary material for: K-RAS Associated Gene-Mutation-Based Algorithm for Prediction of Treatment Response of Patients with Subtypes of Breast Cancer and Especially Triple-Negative Cancer
Source: Cancers (Basel). 2022 Oct 28;14(21):5322. doi: 10.3390/cancers14215322 (PMC9657686; doi:10.3390/cancers14215322)
Supplement: Supplementary file 1 [file cancers-14-05322-s001.zip › cancers-1912723-supplementary.pdf]

Supplementary Data

Supplemental Tables

Table S1. Performance of the 12-Gene Algorithm for distinguishing progression and non-progression after treatment in the TCGA Cohort (*n* = 399), triple-negative subgroup (*n* = 42), and luminal A subgroup (*n* = 155).

	**TCGA Cohort (*n* = 399)**			**Triple-negative Subgroup (*n* = 42)**			**Luminal A Subgroup (*n* = 155)**		
	Positive	Negative	Total	Positive	Negative	Total	Positive	Negative	Total
Progression	33	13	46	145	23	168	375	62	437
Non-progression	12	341	353	3	36	39	16	274	290
Total	45	354	399	148	59	207	391	336	727
Sensitivity (95% CI)		72% (59-85%)			71% (38-105%)			85% (65-104%)	
Specificity (95% CI)		97% (95-98%)			97% (92-103%)			96% (93-100%)	
PPV (95% CI)		73% (60-86%)			83% (54-113%)			69% (46-91%)	
NPV (95% CI)		97% (95-98%)			83% (54-113%)			99% (97-101%)	

CI: confidence interval; PPV: positive predictive value; NPV: negative predictive value.

Table S2. Performance of the 12-Gene Algorithm for distinguishing progression and non-progression after treatment in the MSK Cohort (*n* = 807), triple-negative subgroup (*n* = 75), and luminal A subgroups (*n* = 501).

	**MSK Cohort (*n* = 807)**			**Triple-negative Subgroup (*n* = 75)**			**Luminal A Subgroup (*n* = 501)**		
	Positive	Negative	Total	Positive	Negative	Total	Positive	Negative	Total
Progression	235	80	315	26	3	29	131	48	179
Non-progression	13	479	492	4	42	46	5	317	322
Total	248	559	807	30	45	75	136	365	501
Sensitivity (95% CI)		75% (70-79%)			90% (79-101%)			73% (67-80%)	
Specificity (95% CI)		97% (96-99%)			91% (83-99%)			99% (97-100%)	
PPV (95% CI)		95% (92-98%)			87% (75-99%)			96% (93-99%)	
NPV (95% CI)		86% (83-89%)			93% (86-101%)			87% (83-90%)	

CI: confidence interval; PPV: positive predictive value; NPV: negative predictive value.
